# Lung Cancer Immunotherapy in Transplant Patients and in Patients With Autoimmune Diseases

**DOI:** 10.3389/fonc.2020.568081

**Published:** 2020-11-19

**Authors:** Tomasz Kubiatowski, Marcin Nicoś, Paweł Krawczyk

**Affiliations:** ^1^ Department of Medical Oncology, Center of Oncology of the Lublin Region, Lublin, Poland; ^2^ Department of Pneumonology, Oncology and Allergology, Medical University of Lublin, Lublin, Poland; ^3^ Science for Life Laboratory, Department of Medical Biochemistry and Biophysics, Karolinska Institutet, Stockholm, Sweden

**Keywords:** programmed cell death receptor type 1, PD-1 ligand, cytotoxic T lymphocyte-associated antigen, preexisting autoimmune disease, transplant recipients

## Abstract

The use of immune checkpoint inhibitors (ICIs) delivered great and new possibilities in modern treatment of many types of cancers. This therapy based on blockade of such molecules as CTLA-4 (cytotoxic T lymphocyte-associated antigen), PD-1 (programmed cell death receptor type 1), or PD-1 ligand (PD-L1) brings a new hope for patients with non-small cell lung cancer (NSCLC), melanoma, or head and neck squamous carcinoma. Efficacy of immunotherapy was proven in many clinical trials. Unfortunately, ICIs treatment was not addressed to the patients with preexisting allogeneic transplants or autoimmune diseases mainly due to high risk of transplant rejection, exacerbation of autoimmune diseases, and risk of serious toxicity. However, it is possible to receive anti-tumor response to ICIs treatment avoiding graft rejection by adjusting the immunosuppression. Obviously, it depends on the type of transplants: the use of immunotherapy is usually possible in kidney or corneal recipients, but it could be difficult in patients with liver and heart transplant. Therefore, the development of biomarkers for tumor response and transplant rejection in ICIs treated patients is essential. Data coming from published literature support the possibilities of using ICIs in patients with preexisting autoimmune diseases who undergoing proper management of side effects of immunotherapy or when the potential benefits of such treatment outweigh the potential risks. This depends on the type of autoimmune disease and may be difficult or not feasible in patients with systemic lupus erythematosus or systemic sclerosis. Therefore, it may be appropriate to include cancer patients with preexisting autoimmune disease or with allogeneic transplants in clinical trials using immunotherapy when no other effective cancer treatment options exist.

## Introduction

The treatment of non-small cell lung cancer (NSCLC) is one of the most serious challenges facing modern oncology. It is due to continuous increase in morbidity and mortality caused by this type of cancer. According to the epidemiological data available on the World Health Organization (WHO) websites, in 2040 the estimated increase in the number of lung cancer cases in the world and the number of deaths resulting from this disease will be 72.5% and 76.3%, respectively. These data clearly indicate the need for continuous development of modern therapies that can improve the treatment outcomes and reduce the number of deaths (1). Enormous expectations are associated with the introduction of immune checkpoint-blocking antibodies into routine clinical practice; mainly programmed death receptor 1 (PD-1) or its ligand (PD-L1) (2–5). The published research results have clearly demonstrated the therapeutic value of this form of immunotherapy. It should be kept in mind that clinical studies constituting the basis for the registration of PD-1 or PD-L1 inhibitors usually did not include indications for use in patients with a history of lung cancer concurrent with autoimmune disease or patients after organ transplants in whom the use of immunosuppressive therapy could lead to immune system dysfunction significantly affecting the detection and elimination of cancer cells (1–6). Understanding the mechanisms determining the occurrence of immune toxicity symptoms and concern for the safety of patients during therapy raised a number of questions regarding the possibility of using this form of treatment in patients with concomitant autoimmune diseases. This is all the more important since, according to the literature data, concomitant autoimmune diseases occur in 14%–25% of patients diagnosed with NSCLC (1). The inability to use immunotherapy in this clinical situation would determine the need for the application of chemotherapy or radiation therapy, which would be a suboptimal treatment for many patients. 

A beneficial therapeutic effect resulting from the use of antibodies against PD-1 (nivolumab, pembrolizumab) or PD-L1 (atezolizumab, durvalumab), manifested in longer disease-free or overall survival, was initially demonstrated in patients experiencing cancer progression after the first and subsequent courses of platinum-based chemotherapy. As demonstrated in the CheckMate 017 study, the use of nivolumab in patients diagnosed with squamous cell carcinoma led to longer time to disease progression (3.5 vs. 2.8 months) and overall survival (OS) (9.2 vs. 6.0 months) compared to standard docetaxel-based treatment (2). The use of nivolumab in patients diagnosed with non-squamous cell lung cancer was associated with an increase in OS (12.2 vs. 9.4 months) without affecting progression-free survival (PFS) (2.3 vs. 4.2 months) (3). Slightly different results were obtained in the Keynote-010 study analyzing the efficacy and safety of pembrolizumab at a dose of 2 mg/kg body weight or 10 mg/kg body weight in patients diagnosed with NSCLC experiencing progression after chemotherapy. In this study, the inclusion criteria were PD-L1 protein expression on at least 1% of cancer cells. As demonstrated in the analyzes, the use of pembrolizumab, regardless of dose (2 or 10 mg/kg), was associated with a significant increase in median OS compared to docetaxel (10.4 and 12.7 months for pembrolizumab 2 and 10 mg/kg, respectively, and 8.5 months for docetaxel). The effect of pembrolizumab on the median PFS was observed only in the group of patients with PD-L1 expression on the surface of at least 50% of cancer cells (4).

Understanding the role of PD-1 and PD-L1 interactions in the regulation of anti-tumor response has also initiated a number of studies to determine the effect of using anti–PD-L1 antibodies in the treatment of patients with NSCLC. One of them was the POPLAR study analyzing the efficacy and safety of atezolizumab in NSCLC patients experiencing progression after previous treatment. It has been demonstrated that the use of this antibody, compared to docetaxel, was associated with a significant increase in OS (12.6 vs. 9.7 months), with its therapeutic effect being especially apparent in patients with high percentage of tumor cells with PD-L1 expression (5). The POPLAR study was extended to the OAK phase III study. Compared to docetaxel, atezolizumab was associated with an increase in median OS (13.8 vs. 9.6 months, respectively), the effect being particularly evident in patients with high percentage of tumor cells with PD-L1 expression (mOS 15.7 vs. 10.3 months) and independent of the histological subtype of lung cancer. The benefit of using atezolizumab was also observed in patients without PD-L1 expression on cancer cells (12.6 vs. 8.9 months) (6).

The results of the above studies constitutes the basis for registration of PD-1 or PD-L1 inhibitors for the treatment of NSCLC after the failure of previous therapies based on platinum derivatives, and also inspired a number of studies analyzing the effect of their use in previously untreated patients. One of them was Keynote-024, a randomized Phase III study comparing the benefits of first line therapy with pembrolizumab to platinum-based chemotherapy in patients with advanced NSCLC expressing PD-L1 on the surface of at least 50% of cancer cells. It demonstrated that the use of pembrolizumab as first-line treatment, compared to standard chemotherapy, led to an increase in median PFS (10.4 vs. 6.0 months, HR = 0.50, p < 0.001) and OS (30.0 vs. 14.2 months, HR = 0.63, p = 0.002) (7).

Statistically significant improvement in median PFS or OS following the use of immune checkpoint inhibitors (ICIs) in monotherapy as the first-line or subsequent therapy and striving for the improvement of treatment outcomes gave birth to a series of concepts involving the combination of PD-1 or PD-L1 inhibitor-based immunotherapy and chemotherapy or radiation therapy (8–11). These concepts were based on a few assumptions. The first, that the increase in the expression of tumor antigens released from cancer cells destroyed by chemotherapy should lead to increased activation of the immune response directed against the tumor cells. Another resulted from the observations demonstrating the increase in tumor immunogenicity due to the induction of tumor cell apoptosis as a result of activation of immunogenic cell death resulting from the use of cytostatics. As a result, damage-associated molecular patterns (DAMPs), including calreticulin, occur on the surface of cancer cells, providing a signal for the activation of immune response directed against cancer cells and, as a consequence, the maturation of dendritic cells and activation of cytotoxic T lymphocytes (8).

This concept formed the basis for a number of studies analyzing the effect of combining chemotherapy and immunotherapy based on anti–PD-1 antibodies in the first-line treatment of patients with NSCLC. The use of pembrolizumab in combination with platinum-based chemotherapy as first-line treatment of NSCLC patients with non-squamous cell histology (Keynote-189) led to an increase in the median PFS (9.0 vs. 4.9 months, HR = 0.48) and median OS (22.0 vs. 10.7 months, HR = 0.56), and the observed clinical benefit was independent of PD-L1 expression on tumor cells (9).

The Keynote-407 study analyzed the effect of the combined use of pembrolizumab and chemotherapy in the first-line treatment of patients with advanced squamous-cell NSCLC. The use of combination therapy resulted in prolonged median time to disease progression compared to chemotherapy (6.4 vs. 4.8 months, HR = 0.56) and median OS (15.9 vs. 11.3 months, HR = 0.64), with the therapeutic effect being independent of PD-L1 expression, similarly to Keynote-189 study results (10).

A remarkably interesting concept was the use of immunotherapy in consolidation treatment in patients with locally advanced NSCLC. As demonstrated in the PACIFIC study, the use of durvalumab following radical radio-chemotherapy led to the increase in the median time to distant metastasis (11). Updated results from the study were presented at ASCO 2019 annual meeting. Median OS was not reached (NR; 95% CI, 38.4 months–NR) with durvalumab versus 29.1 months (95% CI, 22.1–35.1) with placebo. The 12-, 24-, and 36-month OS rates with durvalumab and placebo were 83.1% versus 74.6%, 66.3% versus 55.3%, and 57.0% versus 43.5%, respectively (7).

The presented beneficial therapeutic effects resulting from the use of immunotherapy based on checkpoint inhibitors are inextricably linked to the risk of complications following the activation of the immune system. The mechanism of their occurrence is not fully understood. As presented in the study of Postow et al., they may be the result of incorrect recognition of host cell antigens by the activated T cells, an increase in the concentration of antibodies recognizing autoantigens or an increase in the concentration of pro-inflammatory cytokines in tissues (12). The occurrence of immunological side effects in the course of therapy applies to almost all patients receiving PD-1, PD-L1 or CTLA-4 inhibitors, with approximately 15%–30% of patients reaching CTCAE (Common Terminology Criteria for Adverse Events) grade 3 or 4. In most patients, temporary cessation of therapy or the use of immunosuppressive treatment leads to amelioration or complete resolution of immunological toxicity enabling drug re-administration. In some patients, despite the use of steroids or other immunosuppressive medications, it is necessary to discontinue the therapy. This is more common in patients receiving anti–CTLA-4 antibodies than in patients treated with anti–PD-1 or PD-L1 (16% vs. <12%, respectively) (2, 13–18). The combined use of PD-1 and CTLA-4 inhibitors leads to a significantly higher percentage of grade 3 or 4 immune complications (46%–59% of patients) (19). The problem of toxicity of the applied immunotherapy is of particular importance in patients with concomitant autoimmune diseases.

In general, the emergence of autoimmune diseases is the result of disorders in the mechanisms that determine the tolerance of own antigens (8, 12, 19). They may be the consequence of incorrect elimination of autoreactive T lymphocytes during their maturation in the thymus, induction of antigen-specific regulatory T lymphocytes (Tregs), or they may stem from the disturbance in the mechanisms of peripheral tolerance, including antigen sequestration, determining varied immunogenicity of different autoantigen epitopes. Higher expression of proinflammatory cytokines in body tissues leads to increased expression of MHC (major histocompatibility complex) particles, co-stimulatory proteins, proteases, and subsequent host cell presentation of low immunogenicity epitopes recognized by the activated T cells (8). In the context of immunotherapy, the particularly important phenomenon is the lymphocyte anergy resulting from virgin lymphocyte stimulation only by a signal coming from the T cell receptor (TCR) in the absence of co-stimulatory signal coming from the CD28 receptor protein, which in turn can lead to lymphocyte death.

An additional factor inducing the state of anergy is the activation of immune checkpoints (e.g., CTLA-4, PD-1). This is important because stimulation of the PD-1 receptor inhibits the effector function of T cells in tissues, which is crucial for preventing the activation of autoreactive T cells in response to the autoantigen present in the body (8, 12, 19). In addition to maintaining peripheral tolerance, the interaction of PD-1 and PD-L1 plays a role in the selection of lymphocytes in the thymus and in immunologically privileged sites. Therefore, the use of therapy based on PD-1 blocking antibodies allows cytotoxic T lymphocytes to effectively destroy cancer cells at the cost of disrupting the process of self-antigen tolerance (20). Comprehension of these correlations helps explain a number of questions regarding the possibility of using immunotherapy in patients with concomitant autoimmune diseases.

## Possibilities of Using Immunotherapy in Patients With Concurrent Neoplastic and Autoimmune Diseases

ESMO (European Society for Medical Oncology) recommendations published in 2018 allow the use of immunotherapy based on PD-1-, PD-L1-, or CTLA-4-blocking antibodies in selected patients, noting that it may lead to exacerbation of the symptoms of autoimmune disease, requiring the use of immunosuppressive treatment (21). However, there are no study results that directly compare the toxicity resulting from the use of anti–PD-1 and anti–PD-L1 antibodies. However, it is commonly believed that side effects observed in the course of therapy with anti–PD-1 or anti–PD-L1 antibodies are less pronounced than those observed in the group of patients receiving anti–CTLA-4 antibodies or treated with a combination of anti–CTLA-4 and anti–PD-1 antibodies (22). Available literature data come from retrospective analyzes and include heterogeneous groups of patients both in terms of diagnosed cancers, applied immunotherapy, as well as the type of autoimmune disease.

Based on the literature analysis, Abdel-Wahab et al. presented the results of the use of immunotherapy in cancer treatment in 123 patients with concomitant autoimmune diseases presented in 49 publications (23). The dominant diagnosed types of cancer were cutaneous melanoma (83.7%), followed by NSCLC (13%), renal cell carcinoma (2.4%), and Merkel cell carcinoma (0.8%). In addition, 83.5% of patients received treatment for autoimmune disease prior to the introduction of immunotherapy, 46.2% had symptoms of active disease at the start of immunotherapy, and 43.6% required treatment due to symptoms of active autoimmune disease. Most of the analyzed patients received PD-1 inhibitors (52%) as part of their immunological treatment. Ipilimumab was used in 44% of patients included in the analyzes. Symptoms of toxicity related to immunotherapy were found in 75% of patients, with recurrence or exacerbation of concomitant autoimmune disease symptoms in 41% of patients, while 25% of patients had previously unobserved clinical symptoms, the most common of which were colitis (14%) and hypopituitarism (5%). Importantly, there were no differences in the incidence of adverse events associated with immunotherapy in patients with active and inactive autoimmune disease (67% vs. 75%). Exacerbation of autoimmune disease symptoms has been more frequently observed in patients receiving PD-1 or PD-L1 inhibitors than CTLA-4 inhibitors (62% vs. 36%). Whereas, the use of ipilimumab, compared to nivolumab or pembrolizumab, was associated with more frequent occurrence of immune toxicity symptoms, which had not been observed in patients with autoimmune diseases until the start of immune treatment (42% vs. 26%). The occurrence of adverse effects implied the need for high-dose corticosteroids in 62% of patients, which led to clinically significant improvement in the condition of 90% of these patients. In 17% of patients, despite the use of immunosuppressive therapy, it was necessary to discontinue treatment with ICIs. Five patients with autoimmune disease receiving immunotherapy died due to serious adverse events related to the treatment or progression of cancer (23–26). The occurrence and intensification of the toxicity associated with the applied immunotherapy was correlated with the recorded response to treatment. Partial or complete remission of the neoplastic lesions was found in 50% of patients experiencing adverse effects and 35.7% of patients in whom no complications of the applied treatment were reported.

The above observations are consistent with the results presented by Tison et al., who showed exacerbation of autoimmune disease symptoms in 47% of patients receiving immunotherapy, while in 84% of patients these symptoms did not differ from those observed before the introduction of treatment. On the other hand, intensification of the flare phenomenon was observed mainly in patients whose immunosuppressive therapy was completed less than 3 months before the start of immunotherapy (27).

Interesting observations were also provided by Leonardi et al. (1). The authors analyzed the available literature and identified 56 patients with advanced NSCLC receiving PD-1 or PD-L1 inhibitors who had a history of autoimmune diseases associated with inflammatory changes in the joints, skin and subcutaneous tissue as well as endocrine glands or autoimmune colitis. Seven patients had more than one autoimmune disease. At the start of immunotherapy, 10 of the analyzed patients had symptoms of active autoimmune disease and 11 patients were receiving immunosuppressive or immunomodulatory treatment. The exacerbation of the symptoms of autoimmune disease in the course of immunotherapy concerned only some patients and was characterized with low clinical severity, occasionally requiring intravenous corticosteroid use and withholding the cancer treatment. In most patients experiencing the exacerbation of autoimmune disease, no new symptoms resulting from stimulation of the immune system were observed, and none of the patients included in the study needed complete withdrawal of the immunological treatment. Exacerbation of disease symptoms was more frequently observed in patients whose cancer treatment was initiated in the active phase of the autoimmune disease, while the use or absence of immunosuppressive therapy did not significantly affect the severity of autoimmune symptoms after starting anti–PD-1 or anti–PD-L1 therapy (36 % vs. 20%, p = 0.43). Immunological adverse effects resulting from the antineoplastic therapy were present in 38% of patients and were usually of low severity. Grade 3 or 4 was found in 11% of patients, which is a percentage comparable to that observed in clinical studies excluding patients with concomitant autoimmune diseases at the recruitment stage (7%–15%) (1, 13). Only 4 patients required intravenous corticosteroids due to complications. The severity of adverse effects associated with the conducted immunotherapy was the reason for its premature termination in 8 patients (14%), which is a slightly higher percentage than that observed in patients without immune-related diseases (1, 13). Importantly, the use of ICIs in the first or subsequent lines of treatment had no effect on the risk of immune-related complications associated with the therapy or exacerbation of concomitant autoimmune disease. Analyzing the obtained responses, the overall response rate (ORR) of 22% was found in patients with concomitant autoimmune diseases treated with PD-1 or PD-L1 inhibitors, and the frequency of the observed exacerbations of the disease did not correlate with the noted response to immunological treatment (1, 28).

Another analysis, presented by Danlos et al. included 45 patients mainly with either cutaneous melanoma (36 patients) or NSCLC (6 patients), in whom immunotherapy was used to treat cancer despite the presence of autoimmune disease (29). The results of the analyzes were compared with data from 352 patients, with no history of autoimmune diseases, receiving PD-1 or PD-L1 inhibitors for cancer treatment. In result of the applied immunotherapy, the symptoms of immunological toxicity, mainly grade 2 and 3, occurred in 20 patients, whereby in 11 patients these complications were the result of exacerbation of the symptoms of autoimmune disease. The use of corticosteroids led to complete resolution of symptoms in 9 patients and allowed to continue the therapy with anti–PD-1 antibodies in 15 out of 20 patients. Interestingly, in 16 patients with concomitant autoimmune diseases, neither exacerbation of the autoimmune disease symptoms nor the occurrence of toxicity associated with the conducted immunotherapy was observed during the follow-up period (median 5.1 months). In the case of treatment with anti–PD-1 antibodies, the history of autoimmune diseases determined a higher percentage of observed complications (44.4% vs. 29%) and a reduction in median time to treatment-related side effects (5.4 months vs. 13.0 months). Similarly to other analyses, the results obtained by Danlos et al. did not demonstrate impact of autoimmune diseases on OS and ORR in patients receiving treatment with ICIs.

Slightly different results were presented by Cortellini et al. (30). Based on clinical practice data, they showed a significantly higher frequency of complications of any CTCAE (Common Terminology Criteria for Adverse Events) grade associated with immunotherapy in patients whose cancer coexisted with autoimmune disease compared to that observed in the general population (65.9% vs. 39.9%) (30). The rates of grade 3 or 4 toxicities associated with the immunotherapy were not significantly different in both groups. More importantly, the presence of autoimmune disease was also unaffected by the median PFS and OS in result of the use of anti–PD-1 or anti–PD-L1 antibodies.

Due to the retrospective nature and relatively small groups of patients, the above analyzes should be interpreted with great caution. With one possible exception constituted by the analyzes of Weinstock et al., which included a total of 837 patients diagnosed with autoimmune disease receiving PD-1 or PD-L1 inhibitors to treat cancer (31). As shown, only 9% of patients experienced exacerbation of autoimmune disease in result of therapy, while treatment-related symptoms of toxicity occurred in 17% patients, including 3% with grade 3 or higher (31, 32).

Another “limitation” of the presented analyzes is the fact that they were based on data not derived from clinical trials, which may affect the quality of reporting adverse effects associated with immunological treatment or the symptoms of autoimmune disease exacerbation. The duration of immunotherapy and the extent of active follow-up of the patient after the completion of treatment are also important, as they determine the proper identification of distant toxicities associated with the therapy. Effectiveness of immunotherapy is also determined by treatment protocol and line of treatment in which was applied. The use of anti–PD-1, anti–PD-L1, or anti–CTLA-4 antibodies may be a beneficial and relatively safe therapeutic option in selected patients. The decision to start immunotherapy should be made within a multidisciplinary team and should take the dynamics of autoimmune disease and the need for immunosuppressive therapy into consideration. It seems that cancer treatment based on ICIs may be considered in patients, in whom the exacerbation of symptoms associated with the presence of autoimmune disease does not lead to conditions directly threatening the patient’s life and does not require the use of high doses of corticosteroids and other drugs with immunosuppressive effects. As demonstrated by Martinez-Bernal et al., the use of immunotherapy in patients requiring high doses of steroids to control autoimmune symptoms is associated with a worse therapeutic outcome (33). The treatment should be based on PD-1 or PD-L1 inhibitors rather than CTLA-4-blocking antibodies. The combination of PD-1 inhibitors with CTLA-4 inhibitors is not recommended because numerous clinical studies have demonstrated its association with a higher percentage of CTCAE grade 3 and 4 complications (19, 26). Patients diagnosed with autoimmune disease of the nervous system should not be qualified for immunotherapy. The need for high doses of immunosuppressive drugs would imply reduced effectiveness of immunological therapy and may also be associated with greater difficulty in controlling potential immunological toxicity (19). The use of anti–PD-1, anti–PD-L1, or anti–CTLA-4 antibodies in patients with concomitant autoimmune disease is a procedure with a high risk of complications, therefore, it is not recommended to choose this type of treatment as adjuvant therapy.

## Cancer Immunotherapy in Patients After Organ Transplants

Another extremely interesting issue is the possibility of using immunotherapy in the treatment of cancer in patients after organ transplants. The use of immunosuppressive therapy is associated with a significantly increased risk of cancer (34, 35), which is the second most common cause of death in these patients (36). The risk of death in this group of patients is additionally dependent on the severity of the symptoms of immunosuppression toxicity, as well as on the selection of cancer therapy which, due to the risk of transplant rejection, may be suboptimal for a particular cancer treatment. It is also not entirely clear whether rejection of transplanted organs is a direct result of the use of immunotherapy based on PD-1, PD-L1, and CTLA-4 inhibitors or impaired immune response following treatment (37, 38). Moreover, no reliable risk factors for the rejection of the transplanted organ as a result of the applied immunotherapy have been established, nor has the immunotherapy scheme leading to a specific balance between therapeutic benefit and the risk of transplant rejection been described (28).

Despite the significance of the issue, the available literature data are limited and include mostly case reports (39–41) or results of retrospective analysis carried out in small groups of patients. Very interesting data come from analyzes performed by Abdel-Wahab et al. (36). The authors of the study analyzed the result of the use of immunotherapy administered as life-saving treatment in 39 patients with melanoma (62%), cutaneous squamous cell carcinoma (15%), hepatocellular carcinoma (10%), or NSCLC (8%) diagnosed in the course of immunosuppression after kidney (59%), liver (28%), or heart (13%) transplantation. The treatment mainly involved the use of PD-1 inhibitors (77%), while the combination of anti–PD-1 and anti–CTLA-4 antibodies was used in only 3% of patients. The time from transplantation to the introduction of immunological treatment ranged from 1 to 32 years (median 9.0 years). In result of the applied treatment, 16 patients (41%) experienced immunological reactions promoting the rejection of the transplanted organ; the median time from the start of immunotherapy to the onset of the said reactions was 21 days. Despite the reintroduction of immunosuppressive therapy, definitive transplant rejection occurred in 13 of these patients (81%). The percentage of patients with transplant rejection was not correlated with the time since organ transplantation, the type of antibodies used to block the immune checkpoints or the transplanted organ. In 15 (38%) patients included in the analyzes, there were no signs of transplanted organ dysfunction or of toxicity associated with the conducted immunotherapy. Objective responses to the applied treatment were more frequently observed in patients without the initiation of transplant rejection reaction, as well as in those receiving steroids in a dose smaller than equivalent to 10-mg prednisone at the time of the introduction of immunotherapy (36, 39, 40, 42, 43).

Similar results are presented in the work of de Bruyn et al., who analyzed the effect of immunotherapy in patients with liver (19 patients) or kidney (29 patients) transplantation (42). Response to treatment was more frequently observed in patients after kidney transplantation than the ones after liver transplantation (45% and 21%, respectively), and this effect was also associated with slightly more frequent rejection of the transplanted organ (45% vs. 37%). In 21% of patients, the obtained immune response was not accompanied by the activation of immune responses associated with the rejection of the transplanted organ. The safety of ICIs in kidney transplant recipients was assessed by Monohar et al. (43). Based on a review of the literature, they identified 44 kidney transplant recipients diagnosed with melanoma (68%), lung cancer (11%), and metastatic squamous cell carcinoma of the skin (11%), or other malignant neoplasm (9%) treated mainly with nivolumab (24%), pembrolizumab (25%), or ipilimumab (20%). Acute renal allograft rejection was reported in 18 patients (41%). The median time from the initiation of immunotherapy to the diagnosis of acute rejection was 24 days. Twenty-five (59%) patients had no organ rejection. Complete response, partial response or disease stabilization were seen in 4, 5, and 3 patients, respectively. Progressive disease was diagnosed in 14 patients treated with ICI.

Another systemic review was done by the D’Izarny-Gargas group (44). They identified 48 original case reports or short series of 83 solid organ recipients, among whom kidney transplants were performed in 53 patients, liver in 24 patients, and heart transplantation in 6 of them. Skin melanoma was the predominant maligancy (46 patients). The next ones were hepatocellular carcinoma (12 patients) and squamous cell carcinoma of the skin (10 patients). The median time from completing organ transplantation to initiating immunotherapy was 9.3 years. Most patients received anti–PD-1 antibodies (60 patients) in immunological treatment. 13 patients were treated with ipilimumab, and combined anti–PD-1/anti–CTLA-4 therapy was used in 9 cases. Allograft rejection due to immunotherapy was observed in 33 patients. The median time from the initiation of ICIs to the onset of rejection was 5.6 weeks, but in the majority of patients, allograft rejection was noticed within the first 2 weeks of treatment. Median OS was significantly shorter in liver transplant recipients compared to those with kidney or heart transplant (29.0 vs. 36.0 vs. 46.0 weeks, respectively).

As presented above, the rejection of transplanted organs is one of the most frequently occurring immune complications, however, as the literature data show, it can significantly precede the occurrence of other “classic” adverse effects associated with immunotherapy (28).

The introduction of immunotherapy into routine clinical practice has brought a significant breakthrough in the treatment of many cancers, including NSCLC. The development of transplantology and immunosuppressive therapies results in longer survival of patients after organ transplantation and is the reason for the increase in cancer incidence in this group of patients. In every patient, the use of immunotherapy should be preceded by a detailed discussion of all risks associated with the therapy, including those resulting from transplant rejection, and the final decision should be made in consultation with the patient by a multidisciplinary medical team working closely with specialists in the field of transplantation or clinical immunology. Patients with pre-existing autoimmune disease always should be offered with clinical trials. According to clinicalTrials web page there are two trials dedicated for patients with pre-existing autoimmune disease: NCT03656627 (Nivolumab in Treating Patients With Autoimmune Disorders or Advanced, Metastatic, or Unresectable Cancer) and NCT03816345 [A Phase Ib Study of Nivolumab in Patients With Autoimmune Disorders and Advanced Malignancies (AIM-NIVO)].

## Author Contributions

All authors listed have made a substantial, direct, and intellectual contribution to the work. TK wrote the first draft of the manuscript. MN and PK contributed to preparation of the submitted and revised versions of the manuscript. All authors contributed to the article and approved the submitted version.

## Funding

This work was supported by Funds from Medical University of Lublin to PK and a START scholarship from the Foundation for Polish Science (FNP) to MN.

## Conflict of Interest

The authors declare that the research was conducted in the absence of any commercial or financial relationships that could be construed as a potential conflict of interest.

## References

[1] Leonardi GC Gainor JF Altan M Kravets S Dahlberg SE Gedmintas L Safety of programmed death-1 pathway inhibitors among patients with non–small-cell lung cancer and preexisting autoimmune disorders. J Clin Oncol (2018) 36:1905–12. 10.1200/JCO.2017.77.0305 PMC6553840 29746230

[2] Brahmer J Reckamp KL Baas P Crinò L Eberhardt WEE Poddubskaya E Nivolumab versus docetaxel in advanced squamous-cell non-small-cell lung cancer. N Engl J Med (2015) 373:123–35. 10.1056/NEJMoa1504627 PMC4681400 26028407

[3] Borghaei H Paz-Ares L Horn L Spigel DR Steins M Ready NE Nivolumab versus docetaxel in advanced nonsquamous non-small-cell lung cancer. N Engl J Med (2015) 373:1627–39. 10.1056/NEJMoa1507643 PMC5705936 26412456

[4] Herbst RS Baas P Kim D-W Felip E Pérez-Gracia JL Han J-Y Pembrolizumab versus docetaxel for previously treated, PD-L1-positive, advanced non-small-cell lung cancer (KEYNOTE-010): a randomised controlled trial. Lancet (2016) 387:1540–50. 10.1016/S0140-6736(15)01281-7 26712084

[5] Fehrenbacher L Spira A Ballinger M Kowanetz M Vansteenkiste J Mazieras J Atezolizumab versus docetaxel for patients with previously treated non-small-cell lung cancer (POPLAR): a multicentre, open-label, phase 2 randomised controlled trial. Lancet (2016) 387:1837–46. 10.1016/S0140-6736(16)00587-0 26970723

[6] Rittmeyer A Barlesi F Waterkamp D Park K Ciardiello F von Pawel J Atezolizumab versus docetaxel in patients with previously treated non-small- cell lung cancer (OAK): a phase 3, open-label, multicentre randomised controlled trial. Lancet 389:255–65. 10.1016/S0140-6736(16)32517-X PMC6886121 27979383

[7] Reck M Rodríguez-Abreu D Robinson AG Hui R Csoszi T F̈löp A Updated Analysis of KEYNOTE-024: Pembrolizumab Versus Platinum-Based Chemotherapy for Advanced Non-Small-Cell Lung Cancer With PD-L1 Tumor Proportion Score of 50% or Greater. J Clin Oncol (2019) 37(7):537–46. 10.1200/JCO.18.00149 30620668

[8] Leonetti A Wever B Mazzaschi G Assaraf YG Rolfo C Quaini F Molecular basis and rationale for combining immune checkpoint inhibitors T with chemotherapy in non-small cell lung cancer. Drug Resistance Updates (2019) 46:100644. 10.1016/j.drup.2019.100644 31585395

[9] Gadgeel S Rodríguez-Abreu D Speranza G Esteban E Felip E Domine E Updated Analysis From KEYNOTE-189: Pembrolizumab or Placebo Plus Pemetrexed and Platinum for Previously Untreated Metastatic Nonsquamous Non-Small-Cell Lung Cancer. J Clin Oncol (2020) 38(14):1505–17. 10.1200/JCO.19.03136 32150489

[10] Paz-Ares L Luft A Vicente D Tafreshi A Gümüş M Mazeries J Pembrolizumab plus Chemotherapy for Squamous Non-Small-Cell Lung Cancer. N Engl J Med (2018) 379(21):2040–51. 10.1056/NEJMoa1810865 30280635

[11] Antonia SJ Villegas A Daniel D Vicente D Murakami S Hui R Overall survival with durvalumab after chemoradiotherapy in stage III NSCLC. N Engl J Med (2018) 379(24):2342–50. 10.1056/NEJMoa1809697 30280658

[12] Postow MA Sidlow R Hellmann MD Immune-related adverse events associated with immune checkpoint blockade. N Engl J Med (2018) 378:158–68. 10.1056/NEJMra1703481 29320654

[13] Reck M Rodriguez-Abreu D Robinson AG Hui R Csoszi T Fulop A Pembrolizumab versus chemotherapy for PD- L1-positive non-small-cell lung cancer. N Engl J Med (2016) 375:1823–33. 10.1056/NEJMoa1606774 27718847

[14] Wolchok JD Chiarion-Sileni V Gonzalez R Rutkowski P Grob JJ Cowey CL Overall survival with combined nivolumab and ipilimumab in advanced melanoma. N Engl J Med (2017) 377:1345–56. 10.1056/NEJMoa1709684 PMC5706778 28889792

[15] Robert C Schachter J Long GV Arance A Grob JJ Mortier L Pembrolizumab versus ipilimumab in advanced melanoma. N Engl J Med (2015) 372:2521–32. 10.1056/NEJMoa1503093 25891173

[16] Motzer RJ Escudier B McDermott DF George S Hammers HJ Srinivas S Nivolumab versus everolimus in advanced renal-cell carcinoma. N Engl J Med (2015) 373:1803–13. 10.1056/NEJMoa1510665 PMC5719487 26406148

[17] Powles T Duran I van der Heijden MS Loriot Y Vogelzang NJ De Giorgi U Atezolizumab versus chemotherapy in patients with platinum-treated locally advanced or metastatic urothelial carcinoma (IMvigor211): a multicentre, open-label, phase 3 randomised controlled trial. Lancet (2018) 391:748–57. 10.1016/S0140-6736(17)33297-X 29268948

[18] Motzer RJ Tannir NM McDermott DF Frontera A Melichar B Choueiri TK Nivolumab plus ipilimumab versus sunitinib in advanced renal-cell carcinoma. N Engl J Med (2018) 378:1277–90. 10.1056/NEJMoa1712126 PMC5972549 29562145

[19] Kennedy LC Bhatia S Thompson JA Grivas P Preexisting Autoimmune Disease: Implications for Immune Checkpoint Inhibitor Therapy in Solid Tumors. JNCCN (2019) 17(6):750–7. 10.6004/jnccn.2019.7310 31200356

[20] Nowis D Wańkowicz-Kalińska A Winiarska M Zjawiska Autoimmunizacyjne. In: Gołąb J Jakóbisiak M Lasek W Stokłosa T, editors. Immunologia. Warsaw, Poland: Wydawnictwo Naukowe PWN (2018). p. 362–83.

[21] Span L Larkin J Menzies AM Immunotherapy in Special Populations. In: Haanen J Lugowska I Garassino MC Califano R, editors. ESMO handbook of Immuno-oncology. Lugano, Switzerland: ESMOpress (2018). p. 267–77.

[22] Pantuck M McDermott D Drakaki A To Treat or Not to Treat: Patient Exclusion in Immune Oncology Clinical Trials Due to Preexisting Autoimmune Disease. Cancer (2019) 125:3506–13. 10.1002/cncr.32326 31318445

[23] Abdel-Wahab N Shah M Lopez-Olivo MA Suarez-Almazor ME Use of immune checkpoint inhibitors in the treatment of patients with cancer and preexisting autoimmune disease. Ann Intern Med (2018) 169:133–4. 10.7326/L18-0209 30014109

[24] Kamil F Cohen M Kumta N Wan D Ipilimumab-induced colitis in a patient with ulcerative colitis and lung cancer. Am J Gastroenterol (2013) 108:S404. 10.14309/00000434-201310001-01361

[25] Pedersen M Andersen R Nørgaard P Jacobsen S Thielsen P Straten PT Successful treatment with ipilimumab and interleukin-2 in two patients with metastatic melanoma and systemic autoimmune disease. Cancer Immunol Immunother (2014) 63:1341–6. 10.1007/s00262-014-1607-y PMC11028899 25227926

[26] Johnson DB Sullivan RJ Ott PA Carlino MS Khushalani NI Ye F Ipilimumab therapy in patients with advanced melanoma and preexisting autoimmune disorders. JAMA Oncol (2016) 2:234–40. 10.1001/jamaoncol.2015.4368 26633184

[27] Tison A Quere G Misery L Funck-Brentano E Danlos FX Routier E Safety and Efficacy of Immune Checkpoint Inhibitors in Patients with Cancer and Preexisting Autoimmune Disease: A Nationwide Multicenter Cohort Study. Arthritis Rheumatol (2019) 71(12):2100–11. 10.1002/art.41068 31379105

[28] Haanen J Ernstoff MS Wang Y Menzies AM Puzanov I Grivas P Autoimmune diseases and immune-checkpoint inhibitors for cancer therapy: review of the literature and personalized risk-based prevention strategy. Ann Oncol (2020) 31:724–44. 10.1016/j.annonc.2020.03.285 32194150

[29] Danlos FX Voisin AL Dyevre V Michot JM Routier E Taillade L Safety and efficacy of anti- programmed death 1 antibodies in patients with cancer and pre- existing autoimmune or inflammatory disease. Eur J Cancer (2018) 91:21–9. 10.1016/j.ejca.2017.12.008 29331748

[30] Cortellini A Buti S Santini D Perrone F Giusti R Tiseo M Clinical outcomes of patients with advanced cancer and pre-existing autoimmune diseases treated with anti-programmed death-1 immunotherapy: a real-world transverse study. Oncologist (2019) 24(6):e327–37. 10.1634/theoncologist.2018-0618 PMC6656514 30796151

[31] Weinstock C Singh H Maher VE Kim G Pazdur R FDA analysis of patients with baseline autoimmune diseases treated with PD-1/PD-L1 immunotherapy agents. J Clin Oncol (2017) 35:s3018. 10.1200/JCO.2017.35.15_suppl.3018

[32] Remon J Vilariño N Reguart N Immune checkpoint inhibitors in non-small cell lung cancer (NSCLC): Approaches on special subgroups and unresolved burning questions. Cancer Treat Rev (2018) 64:21–9. 10.1016/j.ctrv.2018.02.002 29454155

[33] Martinez-Bernal G Mezquita L Auclin E Ferrara R Planchard D Remon J Baseline corticosteroids (CS) could be associated with absence of benefit to immune checkpoint inhibitors (ICI) in advanced non-small cell lung cancer (NSCLC) patients. Ann Oncol (2017) 28(s5):472. 10.1093/annonc/mdx380.025

[34] Engels EA Pfeiffer RM Fraumeni JF Jr Kasiske BL Israni AK Snyder JJ Spectrum of cancer risk among US solid organ transplant recipients. JAMA (2011) 306(17):1891–901. 10.1001/jama.2011.1592 PMC3310893 22045767

[35] Farrugia D Mahboob S Cheshire J Begaj I Khosla S Ray D Malignancy-related mortality following kidney transplantation is common. Kidney Int (2014) 85(6):1395–403. 10.1038/ki.2013.458 24257690

[36] Abdel-Wahab N Safa H Abudayyeh A Johnson DH Trinh VA Zobniw CM Checkpoint Inhibitor Therapy for Cancer in Solid Organ Transplantation Recipients: An Institutional Experience and a Systematic Review of the Literature. J Immunother Cancer (2019) 7(1):106. 10.1186/s40425-019-0585-1 30992053 PMC6469201

[37] Nankivell BJ Alexander SI Rejection of the kidney allograft. N Engl J Med (2010) 363:1451–62. 10.1056/NEJMra0902927 20925547

[38] Esfahani K Al-Aubodah TA Thebault P Lapointe R Hudson M Johnson NA Targeting the mTOR pathway uncouples the efficacy and toxicity of PD-1 blockade in renal transplantation. Nat Commun (2019) 10(1):4712. 10.1038/s41467-019-12628-1 31624262 PMC6797722

[39] Kittai AS Oldham H Cetnar J Taylor M Immune checkpoint inhibitors in organ transplant patients. J Immunother (2017) 40(7):277–81. 10.1097/CJI.0000000000000180 28719552

[40] Kuo JC Lilly LB Hogg D Immune checkpoint inhibitor therapy in a liver transplant recipient with a rare subtype of melanoma: a case report and literature review. Melanoma Res (2018) 28(1):61–4. 10.1097/CMR.0000000000000410 29140833

[41] Tagliamento M Grossi F Paolino S Rijavec E Genova C Rossi G Nivolumab treatment in advanced lung cancer patient with chronic active hepatitis C and systemic lupus erythematosus. Immunotherapy (2019) 11(10):873–9. 10.2217/imt-2019-0025 31156006

[42] De Bruyn P Van Gestel D Ost P Kruse V Brochez L Van Vlierberghe H Immune checkpoint blockade for organ transplant patients with advanced cancer: how far can we go? Curr Opin Oncol (2019) 31(2):54–64. 10.1097/CCO.0000000000000505 30694841

[43] Manohar S Thongprayoon C Cheungpasitporn W Markovic SN Herrmann SM Systematic Review of the Safety of Immune Checkpoint Inhibitors Among Kidney Transplant Patients. Kidney Int Rep (2019) 5(2):149–58. 10.1016/j.ekir.2019.11.015. Published 2019 Dec 7. PMC7000848 32043028

[44] d’Izarny-Gargas T Durrbach A Zaidan M Efficacy and tolerance of immune checkpoint inhibitors in transplant patients with cancer: A systematic review. Am J Transplant (2020) 20(9):2457–65. 10.1111/ajt.15811 32027461

